# Bioinformatics analysis of ferroptosis-related biomarkers and potential drug predictions in doxorubicin-induced cardiotoxicity

**DOI:** 10.3389/fcvm.2025.1566782

**Published:** 2025-04-24

**Authors:** Jian Yu, Jiangtao Wang, Xinya Liu, Cancan Wang, Li Wu, Yuanming Zhang

**Affiliations:** ^1^ Department of Cardio-Oncology, Tumor Hospital of Xinjiang Medical University, Urumqi, China; ^2^ Xinjiang Medical University, Urumqi, China; ^3^ Department of Cardiothoracic Surgery, General Hospital of Xinjiang Military Command, Urumqi, China; ^4^ Pathology Department, Tumor Hospital of Xinjiang Medical University, Urumqi, China

**Keywords:** doxorubicin-induced cardiotoxicity, ferroptosis, KLHDC3, NDRG1, anisomycin

## Abstract

**Background:**

Doxorubicin-induced cardiotoxicity (DIC) significantly impacts the survival and prognosis of cancer patients. Ferroptosis is involved in the pathogenesis of DIC, but its specific mechanisms remain unclear. This study aims to explore key genes of ferroptosis in DIC and potential therapeutic drugs using various bioinformatics methods.

**Methods:**

This study obtained the GSE106297 and GSE157282 datasets from the GEO database, conducted differential gene expression screening and GSEA enrichment analysis using R software. Subsequently obtained ferroptosis-related genes from FerrDb V2, Genecards, Geneontology, and GSEA databases, performed GO and KEGG enrichment analysis after intersecting them with the differentially expressed genes using a Venn diagram. Utilized LASSO regression, SVM-RFE, and RF algorithms to identify key genes, followed by validation using external datasets (GSE232331, GSE230638) and ROC curve plotting to determine the diagnostic value of key genes. Further validation of the expression levels of key genes were conducted through the establishment of a cell damage model. Constructed an mRNA-miRNA-lncRNA network diagram, and performed immune cell composition analysis using CIBERSORT. Finally, predicted potential drugs for key genes using the DSigDB database.

**Results:**

We obtained 119 genes after intersecting 1380 Differentially Expressed Genes (DEGs) with Ferroptosis-Related Genes (FRGs). Three key genes (KLHDC3, NDRG1, SPHK1) were identified through further analysis using LASSO, SAM-RFE and RF. The ROC analysis demonstrated that KLHDC3 and NDRG1 have significant diagnostic value, and qRT-PCR verification results also showed statistical significance. We constructed miRNA-lncRNA networks by identifying target miRNAs for KLHDC3 (hsa-miR-24-3p, hsa-miR-486-3p, hsa-miR-214-3p) and NDRG1 (hsa-miR-4510, hsa-miR-182-5p, hsa-miR-96-5p). Immunoinfiltration analysis revealed the relationship between KLHDC3, NDRG1 and immune cells. Anisomycin emerges as a promising small molecule drug for treating DIC, exhibiting good relative binding with KLHDC3 and NDRG1.

**Conclusion:**

KLHDC3 and NDRG1 serve as ferroptosis biomarkers implicated in DIC and demonstrate good diagnostic value. In addition, anisomycin may also be a potential drug for treating DIC.

## 1 Introduction

Cancer remains the leading cause of mortality in humans. Despite the advancement of treatment modalities including radiation therapy, chemotherapy, targeted therapy and immunotherapy, the survival outcomes for cancer patients has been rising. However, the toxic side effects associated with these treatments can significantly impair patients’ quality of life (1). Doxorubicin (DOX) belongs to the anthracycline class of broad-spectrum antitumour drugs. It effectively inhibits the progression of various cancers including lymphoma, breast cancer and lung cancer, thereby significantly enhancing long-term survival rates. However, its therapeutic process inevitably leads to cardiotoxicity (2). Doxorubicin-induced cardiotoxicity (DIC) has led to cardiovascular diseases of varying severity in cancer patients receiving this drug. One study indicated that approximately 14%–49% of lymphoma patients developed cardiovascular diseases during DOX treatment, and this incidence is dose-dependent (3, 4). The primary clinical manifestations of DIC include arrhythmia, myocardial injury and cardiac insufficiency. Fatal arrhythmia, in particular, can precipitate serious episodes of sudden cardiac death, thereby limiting the utility of DOX to some extent (5, 6).

The pathogenesis of DIC is multifaceted, involving inflammation, oxidative stress, mitochondrial dysfunction, aberrant intracellular calcium regulation, altered energy metabolism, extracellular matrix remodeling, apoptosis and dysregulation of specific signaling pathways (7). The rapid advancements in bioassay technology enable a deeper comprehension of the pathophysiological processes underlying DIC and elucidating the mechanisms underlying the onset and progression of cardiotoxicity. This understanding offers a theoretical framework for the clinical prevention and treatment of DIC.

Ferroptosis, a unique mode of cell death, exhibits distinctions from both apoptosis and necrosis. It is primarily triggered by abnormal intracellular accumulation of free iron and lipid peroxidation, resulting in oxidative damage to cellular biomolecules and ultimately leading to cell demise (8, 9). Previous studies have shown that ferroptosis plays a crucial role in the pathogenesis of cardiovascular disease and cancer. Nevertheless, the precise mechanisms, signaling pathways and biomarkers implicated in ferroptosis in DIC remain poorly understood (10, 11).

We further investigated the expression levels of ferroptosis-related genes (FRGs) in DIC using bioinformatics methods and machine learning algorithms. This approach allowed us to gain a deeper understanding of the molecular mechanisms underlying the disease (12). Expression data of DOX and control (CON) were obtained from the GEO database, while FRGs were collected from FerrDb V2, GeneCards, Geneontology and GSEA databases. Integrated bioinformatics, enrichment analysis and immune infiltration analysis were performed to identify common ferroptosis biomarkers and assess immune cell infiltration in DIC, providing updated insights into its pathophysiology.

## 2 Materials and methods

### 2.1 Acquisition and processing of data sets

To comprehensively explore the DOX dataset, we accessed the NCBI Gene Expression Omnibus database (http://www.ncbi.nlm.nih.gov/geo/) and retrieved the expression data for DOX and CON, primarily including GSE106297, GSE157282, GSE232331 and GSE230638. GSE106297, based on platform GPL11154, consisted of 4 DOX samples derived from human induced pluripotent stem cell-derived cardiomyocytes (hiPSC-CMs) and 4 CON samples. GSE157282, utilizing platform GPL24676, contained 3 DOX samples from hiPSC-CMs and 3 CON samples. To validate key genes, we utilized two external datasets: GSE232331 and GSE230638. GSE232331, generated on GPL18573, comprised 3 DOX samples from hiPSC-CMs and 3 CON samples. GPL24676 served as the platform for GSE230638, which included 4 DOX samples from hiPSC-CMs and 4 CON samples. Integration of GSE106297 and GSE157282 datasets was performed using R software (version 4.2.1), and batch effects were eliminated using the combat function of the “SVA” package. Principal component analysis (PCA) was employed for dimensionality reduction of expression data from all samples in each gene chip group. Subsequently, the distribution and dispersion of samples after dimensionality reduction were visualized on a two-dimensional coordinate plane to validate the grouping of DOX and CON samples. The selected datasets possess complete gene expression profiles and do not involve ethical, moral, or other conflicts of interest.

### 2.2 Screening for differentially expressed genes (DEGs)

After normalisation of the data, DEGs were analysed in 7 DOX and 7 CON samples using the “limma” package. *P* < 0.05 and |LogFC|>1 were used as the thresholds for DEGs, and the “ggplot2” and “ComplexHeatmap” packages were used to draw volcano maps and heat maps for DEGs.

### 2.3 Acquisition of FRGs

Utilizing “ferroptosis” as the search term, we retrieved 912 FRGs from the Genecards database (https://www.genecards.org/), 535 FRGs from the FerrDb V2 database (http://www.zhounan.org/ferrdb/), 8 FRGs from the Geneontology database (https://geneontology.org/), and 64 FRGs from the GSEA database (https://www.gsea-msigdb.org/gsea/msigdb/). Following merging and deduplication, a total of 1,198 unique FRGs were obtained.

### 2.4 Acquisition of DEGs and FRGs (DE-FRGs) intersection genes

The “VennDiagram” and “ggplot2” packages were utilized for analyzing and visualizing the unique and shared components of DE-FRGs.

### 2.5 Enrichment analysis

To further explore the biological significance of DEGs, we conducted GSEA enrichment analysis using the “clusterProfiler” package and visualized the results using “ggplot2”. Additionally, we conducted Kyoto Encyclopedia of Genes and Genomes (KEGG) and Gene Ontology (GO) enrichment analyses of DE-FRGs using the Srplot website (https://bioinformatics.com.cn/login/). We selected the species “human” and enriched KEGG and GO terms based on their *P*-values, including Biological Process (BP), Cellular Component (CC) and Molecular Function (MF).

### 2.6 Identification of key genes

We identified key genes among the DE-FRGs using three machine learning algorithms: Least Absolute Shrinkage and Selection Operator (LASSO), Support Vector Machine Recursive Feature Elimination (SVM-RFE) and Random Forest (RF). These algorithms were implemented using the “glmnet”, “e1071” and “randomforest” packages, respectively. The results from each algorithm were then intersected to determine the key genes. Expression levels of these key genes were validated by observing the levels of DOX and CON in both training and validation sets, with a significance level set at *P* < 0.05. Finally, the diagnostic value of the key genes was assessed using the receiver operating characteristic curve (ROC curve) and calculating the area under the curve (AUC) in human subjects.

### 2.7 Cell culture and treatment

H9c2 cells, obtained from the Chinese Academy of Sciences Cell Bank (Shanghai, China), were propagated in DMEM high glucose medium, fortified with 10% fetal bovine serum and 1% double antibodies. Cells were incubated at 37°C in an atmosphere containing 5% CO_2_. Upon reaching 80%–90% confluence, cells were passaged. Only those in the logarithmic growth phase and in good condition were selected for further experimentation. H9c2 cells were allocated into a control group (CON) and a doxorubicin-treated group (DOX). Subsequently, cells in each group were subjected to varying concentrations of DOX (0, 0.2, 0.5, 1, 5, 10, 20μM) sourced from MedChemExpress (New Jersey, US). For subsequent experiments, the DOX concentration approximating the IC50 value was employed.

### 2.8 CCK-8 assay

H9c2 cells, adjusted to a density of 3 × 10^3^ cells/ml, were seeded in a 96-well plate. Each group included at least five replicates. Post cell adhesion, they were exposed to varying concentrations of DOX for 24 h, 48 h, and 72 h, respectively. Following this, 10 ul of CCK-8 reagent (AbMole, Houston, US) was added and incubated for 2 h. Finally, cell viability was assessed by measuring the absorbance at 450 nm in each well using a microplate reader.

### 2.9 qRT-PCR

Trizol reagent (15596018, Invitrogen, California, US) was used to extract total RNA from H9c2 cells. RNA was reverse-transcribed into cDNA using the PrimeScript™ RT reagent Kit with gDNA Eraser (RR047A, TakaRa Bio, Kyoto, Japan). The cDNA was quantified using TB Green® Premix Ex Taq™ Green I (RR820A, TakaRa Bio, Kyoto, Japan), following all procedures as per the instructions provided by the reagent kits. The reaction was conducted under the following conditions: 95°C for 15 s, 60°C for 30 s, and 72°C for 30 s, across 40 cycles. Table 1 lists the primers synthesized by Tsingke Biotechnology Co., Ltd. GAPDH (F: AGAAGGCTGGGGCTCATTTG, R: AGGGGCCATCCACAGTCTTC) served as an internal reference, with relative expression calculated using the 2'-ΔΔCt method.

**Table 1:** Primer sequence.

Genes	Primers
KLHDC3	F: 5′-TGCTGTATTGTTGGTGATAAGATTGTC -3′
	R: 5′- GAAGTCCAAGATGTGTAAGTCAGAATG-3′
NDRG1	F: 5′-TACCGCCAGCACATCCTCAAC-3′
	R: 5′-GCCGCTCAATCTCCAGGTCTC-3′

### 2.10 Construction of mRNA-miRNA-lncRNA interaction network

We identified miRNAs targeting the key genes by querying four online databases: miRDB (http://mirdb.org/miRDB), miRWalk (http://mirwalk.umm.uni-heidelberg.de/), TarBase (https://dianalab.e-ce.uth.gr/tarbasev9/interactions) and TargetScan (http://www.targetscan.org/vert_80/). StarBase v2.0 (https://starbase.sysu.edu.cn/) was employed to analyze miRNA-lncRNA interactions, and mRNA-miRNA-lncRNA interaction networks were constructed using Cytoscape version 3.9.1. Furthermore, with the criteria set to “human, CLIP-Data >5” in StarBase v2.0, we utilized the “ggplot2” and “ggalluvial” packages to generate a Sankey diagram illustrating miRNA-lncRNA interactions.

### 2.11 Analysis of immune cell infiltration

The gene expression matrix data were inputted into CIBERSORT to filter samples with *P* < 0.05. Subsequently, the immune cell infiltration matrix was generated and visualized using the “ggplot2” package to depict differences in immune cell infiltration. To investigate the correlation between key genes and immune cell levels, Spearman's rank correlation analysis was employed, and the results were presented using bar graphs created with the “ggplot2” package.

### 2.12 Drug prediction and molecular docking

Drug molecules interacting with key genes were identified via the DSigDB database (http:/ dsigdb. tanlab. org DSigDB Bv1.0/) by selecting the “Diseases/Drugs” module in the Enrichr platform. Adjusted *P*-values < 0.05 were considered statistically significant. To validate the binding affinity between active compounds and key targets, molecular docking was conducted. Compound structure files were obtained from the PubChem website (https://pubchem.ncbi.nlm.nih.gov/), and the SDF files were converted to PDB files using Open Babel software (version 2.3.2). Receptor proteins were retrieved from the UniProt database. Initially, the receptor proteins underwent processes such as desalting and ligand removal using PyMOL. Subsequently, AutoDockTools software was used for further modifications of the receptor proteins, including hydrogenation and charge balancing. Finally, both the receptor proteins and ligand small molecules were converted into pdbqt format. Molecular docking of receptor proteins to ligand small molecules was performed using AutoDock Vina, and the docking results were analyzed in PLIP (https:/ plip- tool. biotec. tu- dresd en. de plip- web plip/index) and visualized using PyMOL.

### 2.13 Statistical analysis

The data underwent statistical analysis using R software (version 4.2.1). Two-sample t-tests were employed for comparisons between groups with normal distributions; otherwise, the Wilcoxon rank sum test was utilized. All statistical findings were visualized and processed using R software, with statistical significance set at *P* < 0.05.

## 3 Results

### 3.1 Data processing and acquisition of DEGs

**Figure 1:**
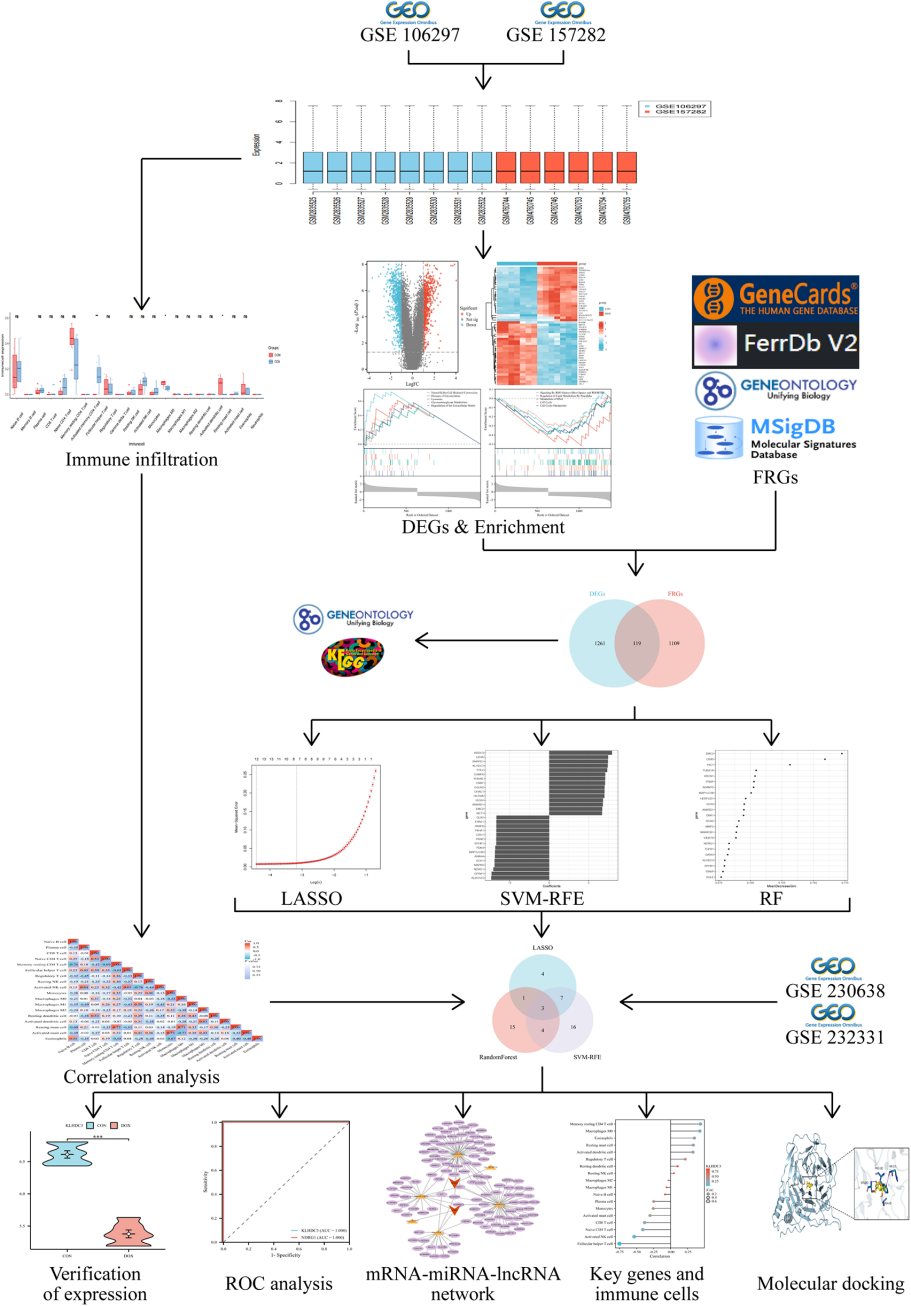
Flowchart of the bioinformatics analysis.

**Figure 2:**
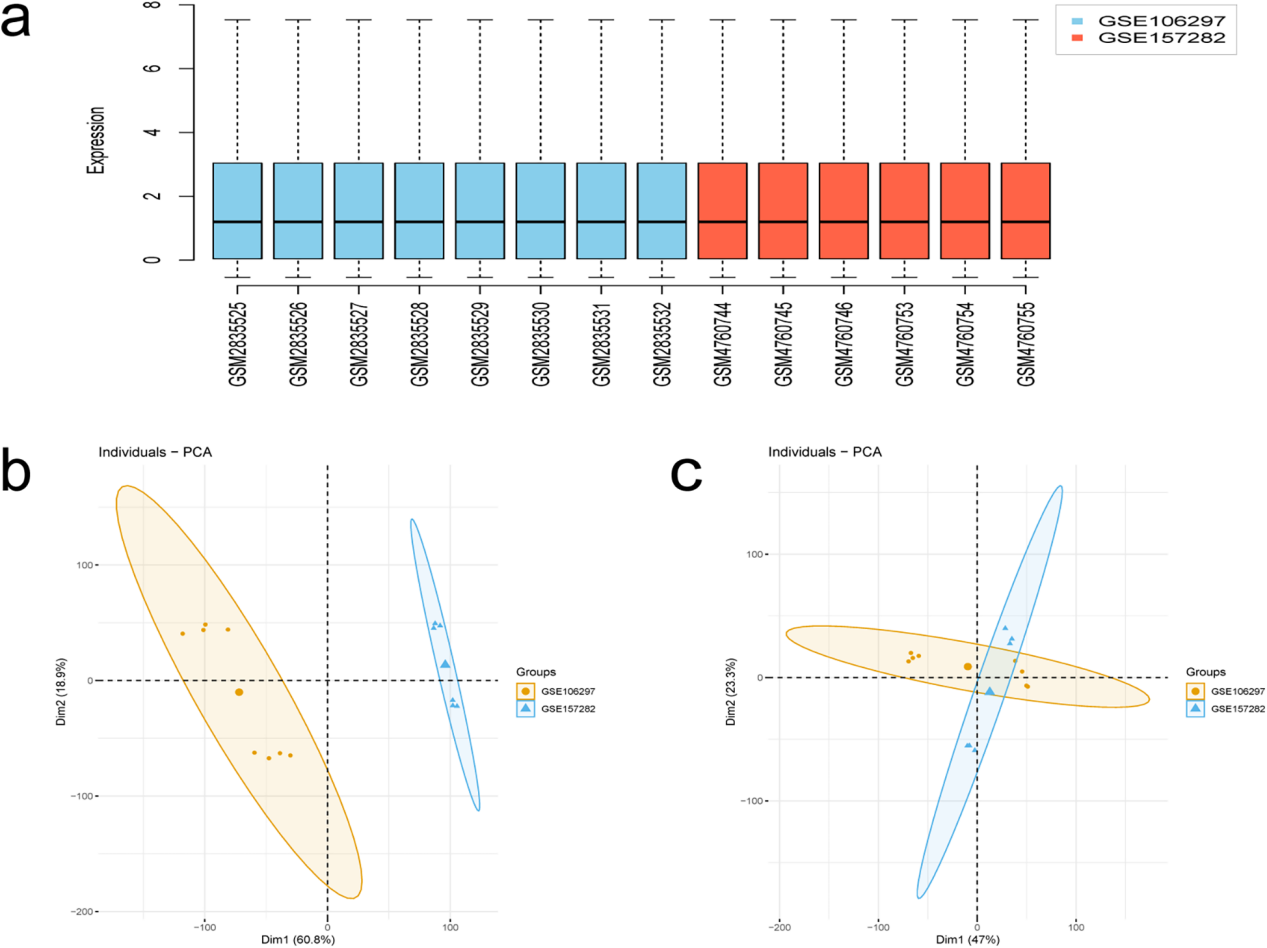
Results of data processing. **(a)** Box-plot of GSE106297 and GSE157282 samples. **(b,c)** PCA analysis.

**Figure 3:**
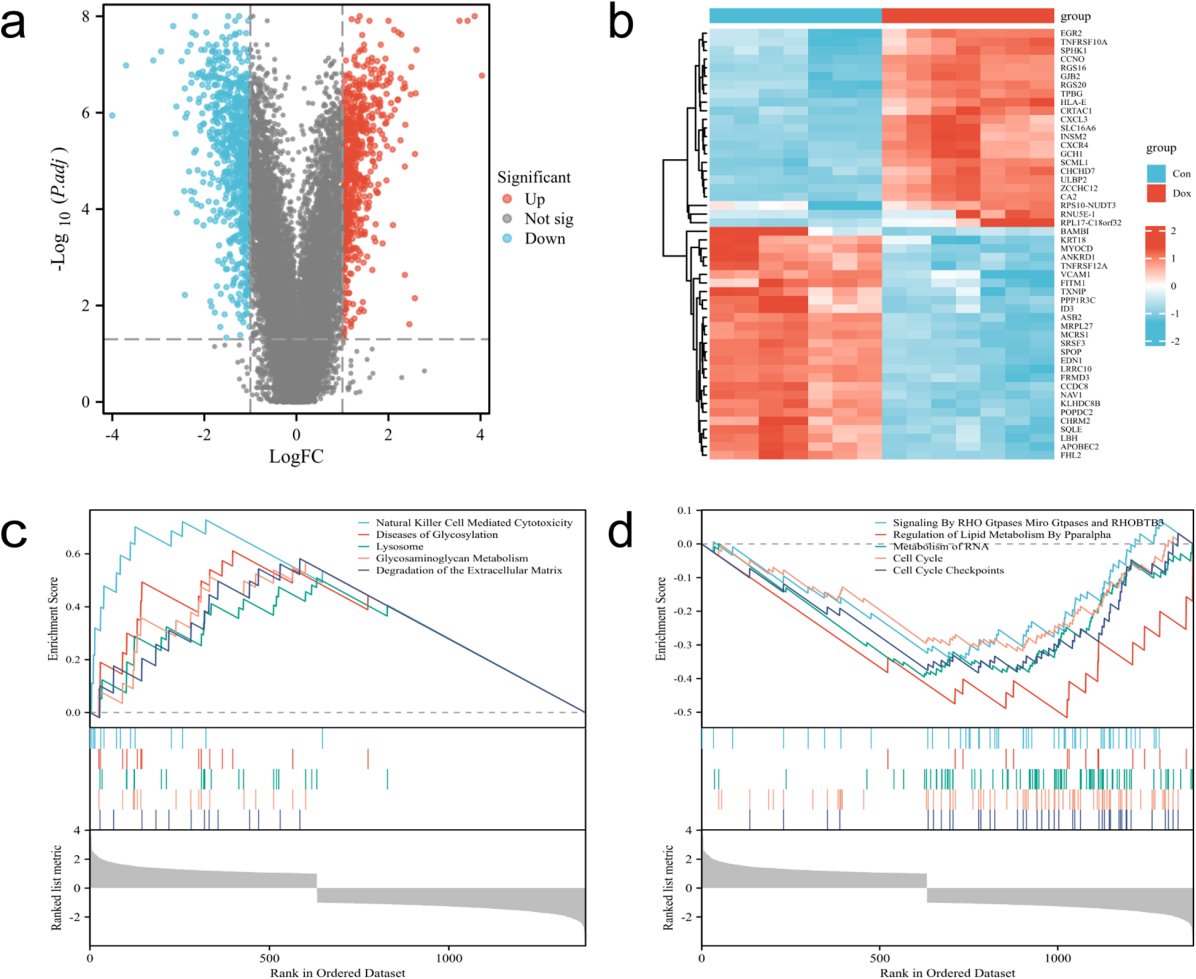
Identification and enrichment analysis of DEGs. **(a)** Volcano plot of DEGs, with red dots indicating upregulated genes and blue dots indicating downregulated genes. **(b)** Heatmap of DEGs. **(c,d)** Enrichment analysis-based upregulated and downregulated pathways determined by GSEA.

The flowchart of this study is shown in Figure 1. After merging and standardizing the GSE106297 and GSE157282 datasets, the results indicate a linear distribution of gene expression data for each sample, suggesting stability (Figures 2a–c). Using the “limma” package, a total of 1,380 DEGs were obtained from the analysis of 7 DOX and 7 CON samples, with 632 upregulated and 748 downregulated DEGs (Figure 3a). The heatmap displays the top 50 upregulated and downregulated DEGs ranked by expression levels (Figure 3b).

### 3.2 GSEA enrichment analysis

The results of GSEA enrichment analysis for DEGs reveal enriched biological processes, primarily showing significant upregulation in processes such as Natural killer cell-mediated cytotoxicity, Diseases of Metabolism, Lysosome, Glycosaminoglycan Metabolism, Degradation of the Extracellular Matrix, while processes like Signaling By RHO Gtpases Miro Gtpases and RHOBTB3, Regulation of Lipid Metabolism By Pparalpha, Metabolism of RNA, Cell cycle, Cell cycle Checkpoints are significantly downregulated (Figures 3c,d).

### 3.3 Acquisition and enrichment analysis of DE-FRGs

**Figure 4:**
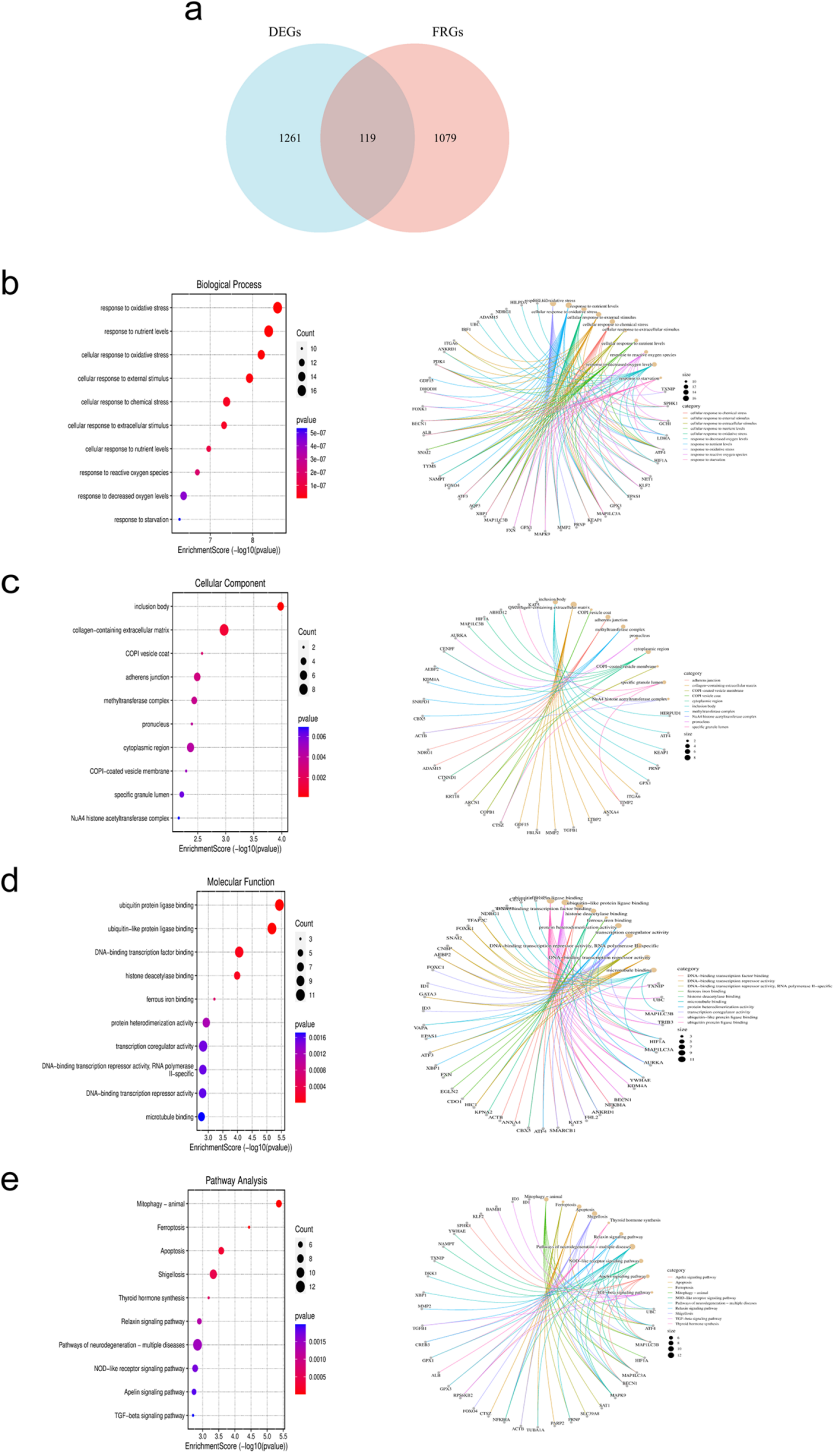
Acquisition and enrichment analysis of intersecting genes between DEGs and FRGs. **(a)** Venn diagram of DE-FRGs. **(b–d)** GO analysis. **(e)** KEGG analysis.

After intersecting DEGs with FRGs, a total of 119 intersecting genes were obtained (Figure 4a). The figure displays the top 10 enriched results. In terms of Biological Processes (BP), the results mainly indicate positive regulation of cellular and organismal responses to oxidative stress, apoptosis processes, negative regulation of intracellular signal transduction, and cellular responses to growth factor stimulation (Figure 4b). In terms of Cellular Component (CC), the results indicate associations with the nucleoplasm, centrosome, adherens junction, transcription regulatory complex, and extracellular matrix containing collagen (Figure 4c). In terms of Molecular Function (MF), the results indicate associations with DNA-binding transcription factor binding, ubiquitin protein ligase binding, protein sequestering activity, protein heterodimerization activity, oxidoreductase activity, and cell adhesion molecule binding (Figure 4d). The KEGG analysis shows associations with pathways such as the cell cycle, ferroptosis, apoptosis, Nod-like receptor signaling pathway, TGF-β signaling pathway, and Th17 cell differentiation (Figure 4e).

### 3.4 Identification of key genes for DE-FRGs

**Figure 5:**
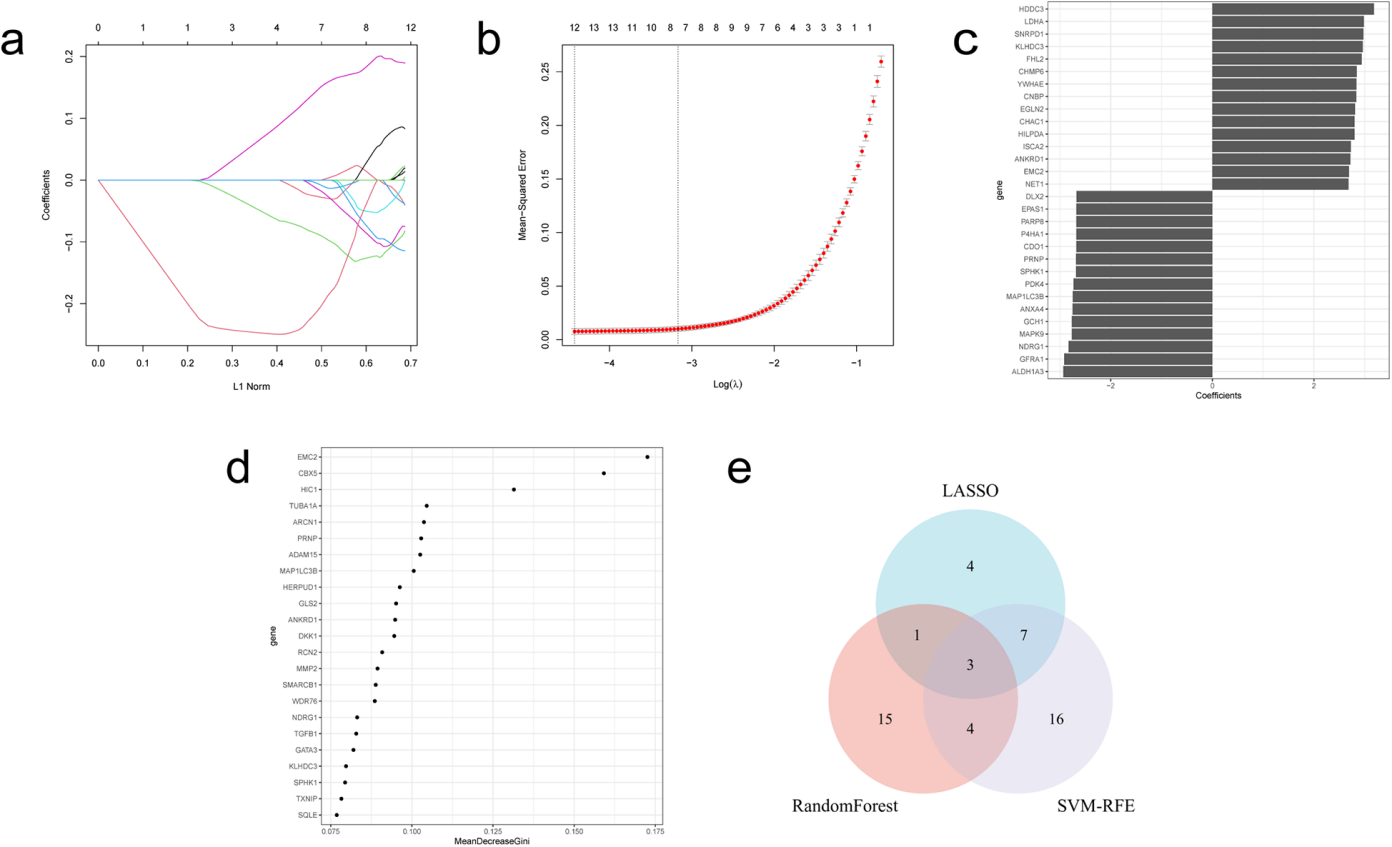
Acquisition of key genes in DE-FRGs. **(a)** LASSO coefficient path. **(b)** LASSO cross validation curve. **(c)** SVM-RFE algorithm. **(d)** Evaluation of random forest error rate as a function of the number of classified trees. **(e)** Venn diagram illustrating the three hub genes derived from the intersection of results obtained from LASSO, SVM-RFE and RF.

**Figure 6:**
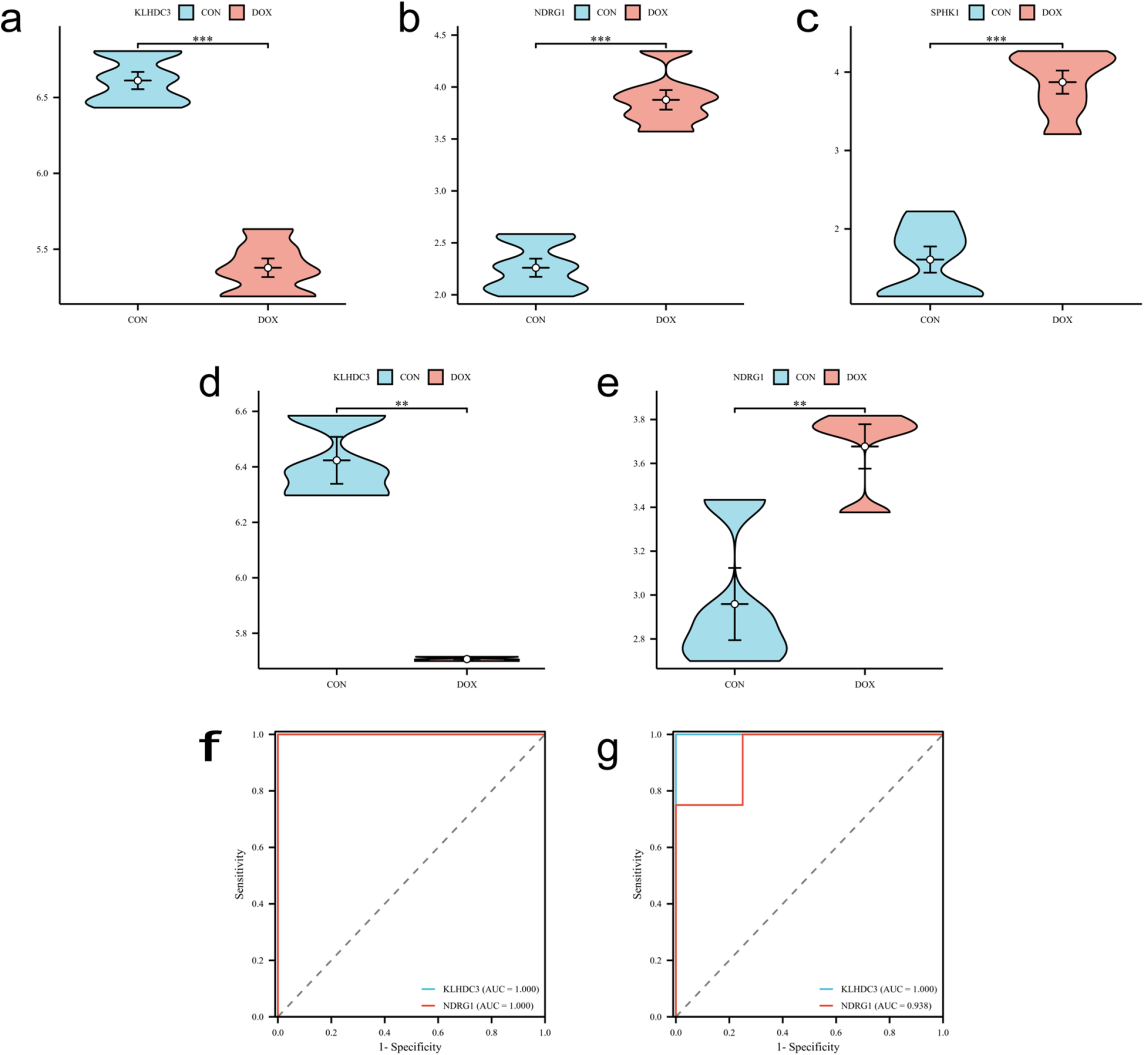
Validation of key genes. **(a–c)** Expression levels of key genes KLHDC3, NDRG1 and SPHK1 in the training set. **(d,e)** Expression levels of key genes KLHDC3 and NDRG1 in the validation set. **(f,g)** ROC curves and AUC values assessing the diagnostic significance of key genes in both the training and validation sets. *P < 0.05, **P < 0.01, ***P < 0.001.

The LASSO regression algorithm, SVM-RFE algorithm and RF algorithm were used to respectively determine 15, 30, 23 genes as candidate feature genes for key genes. To obtain more robust key genes, we further intersected the sets using a Venn diagram and obtained three genes: KLHDC3, NDRG1 and SPHK1 (Figures 5a–e). We validated the expression levels of these key genes in external datasets (GSE232331, GSE230638) and found significant differences in the expression of KLHDC3 and NDRG1 between the DOX and CON groups (*P* < 0.05, Figures 6a–e). By plotting ROC curves, we found that KLHDC3 and NDRG1 have high diagnostic value (AUC = 1), and we used this for subsequent analysis (Figures 6f,g).

### 3.5 The effect of different concentrations of DOX on the viability of H9c2 cells

**Figure 7:**
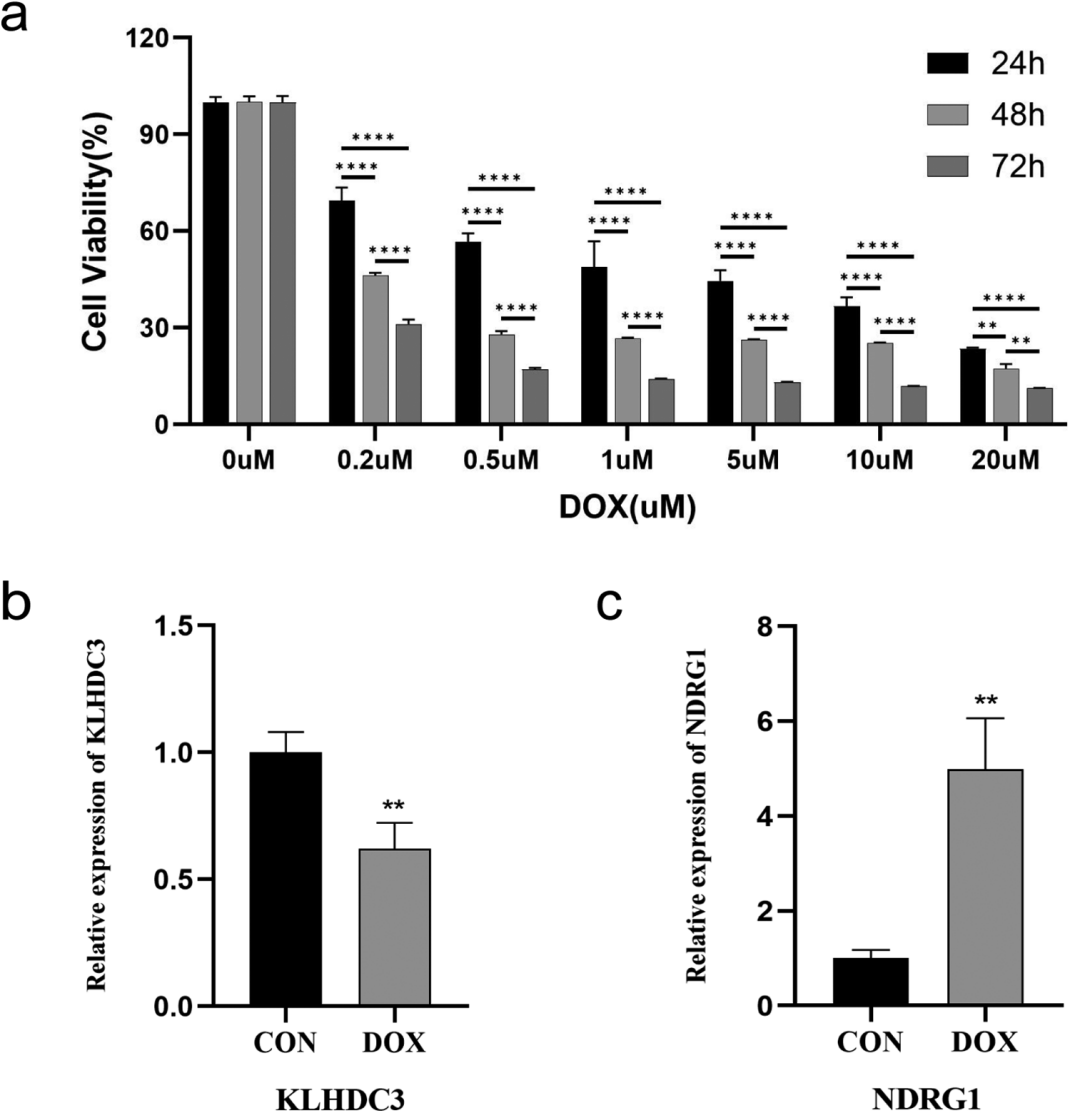
Establishment of H9c2 cell damage model and validation of key genes. **(a)** Cell viability after DOX intervention at different concentrations for 24 h, 48 h, and 72 h, detected by CCK-8. **(b)** Expression level of KLHDC3. **(c)** Expression level of NDRG1. **P < 0.01, ****P < 0.001.

H9c2 cells were treated with varying concentrations of DOX, and cell viability was assessed using the cell counting kit-8 (CCK-8) method to investigate the degree of DOX-induced cardiomyocyte damage. The results revealed that, compared to the control group, the number of H9c2 cells treated with DOX decreased in a dose- and time-dependent manner after 24 h, 48 h, and 72 h. Furthermore, a negative correlation was observed between DOX concentration and cell viability (*P* < 0.01 or *P* < 0.001, Figure 7a).

### 3.6 The expression levels of key genes

A model of cardiomyocyte damage was developed by incubating H9c2 cells with a DOX concentration near the IC50 value (1.5 μM) for 24 h. Following this, quantitative real time polymerase chain reaction (qRT-PCR) was utilized to validate the expression levels of key genes. The results indicated a statistically significant difference in the expression levels of KLHDC3 and NDRG1 between the DOX and CON groups (*P* < 0.05 or *P* < 0.01, Figures 7b,c).

### 3.7 Construction of mRNA-miRNA-lncRNA interaction network

**Figure 8:**
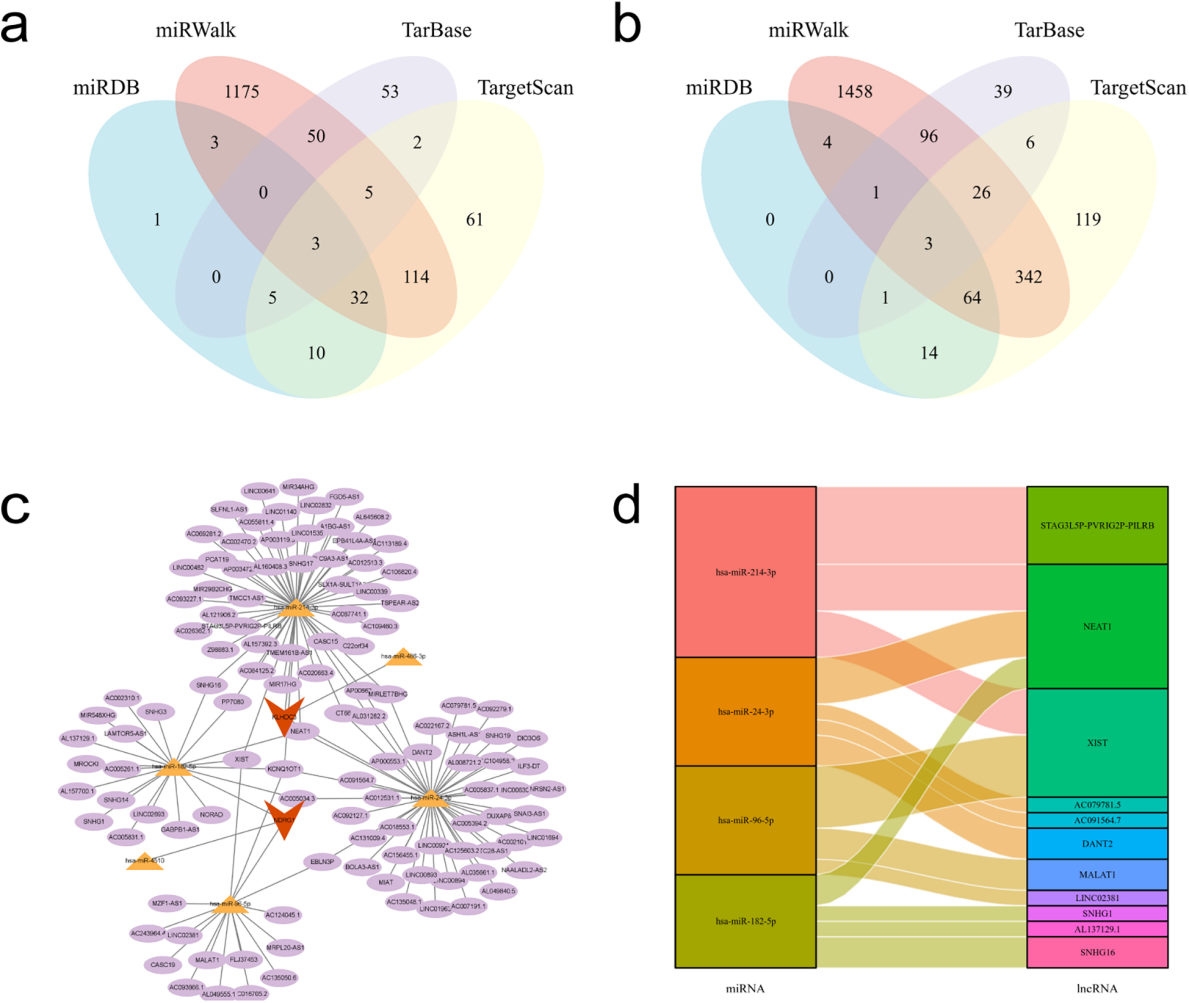
mRNA-miRNA-lncRNA interaction network. **(a,b)** Venn diagrams showing the intersecting target miRNAs for KLHDC3 and NDRG1. **(c)** mRNA-miRNA-lncRNA interaction network constructed using Cytoscape. Key genes are represented in orange, miRNAs in yellow, and lncRNAs in purple. **(d)** Sankey diagram illustrating the miRNA-lncRNA interactions.

The target miRNAs for KLHDC3 are hsa-miR-24-3p, hsa-miR-486-3p and hsa-miR-214-3p (Figure 8a), while the target miRNAs for NDRG1 are hsa-miR-4510, hsa-miR-182-5p and hsa-miR-96-5p (Figure 8b). To explore the target lncRNAs of the above-mentioned miRNAs, we searched for the targets of hsa-miR-24-3p, hsa-miR-486-3p, hsa-miR-214-3p, hsa-miR-4510, hsa-miR-182-5p and hsa-miR-96-5p using StarBase v2.0. Subsequently, we constructed a miRNA-lncRNA network diagram for KLHDC3 and NDRG1 (Figure 8c). Additionally, using “human, CLIP-Data >5” as the criteria in StarBase v2.0, we generated a Sankey diagram (Figure 8d).

### 3.8 Immune infiltration analysis

**Figure 9:**
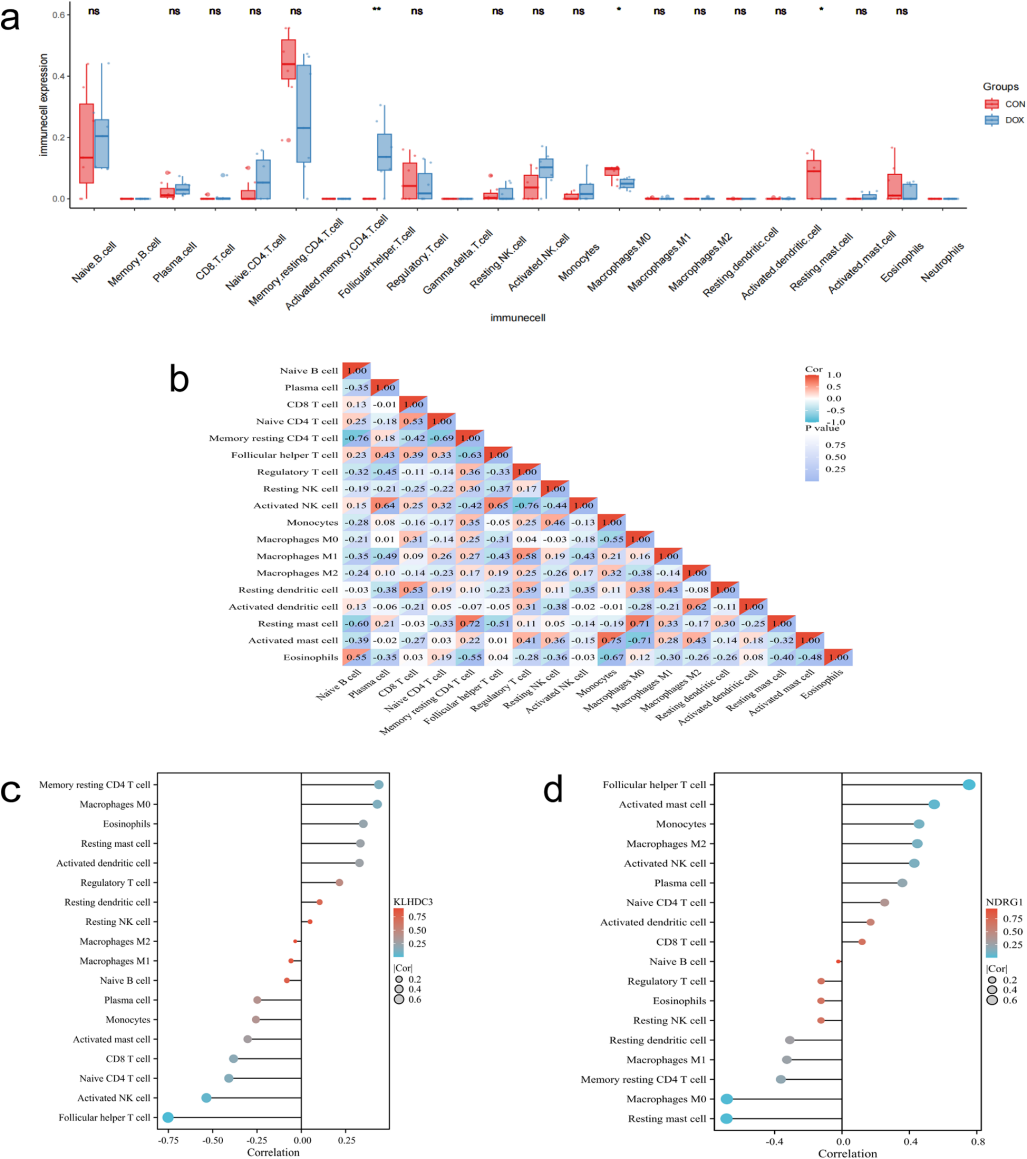
Immune infiltration analysis. **(a)** Box plot displaying the proportions of various immune cells. **(b)** Correlation analysis of different immune cells. **(c,d)** Bar chart depicting the correlation between key genes and immune cells. *P < 0.05, **P < 0.01, ***P < 0.001.

The Figure 9a displays the proportions of 22 immune cells in the DOX and CON groups. The differences between the two groups in terms of Follicular helper T cells, Macrophages M0, and Resting mast cells are statistically significant (*P* < 0.05). The correlation analysis results indicate that the strongest negative correlation exists between Memory resting CD4 T cells and Native B cells, as well as between Activated NK cells and Regulatory T cells (R = −0.76). The strongest positive correlation is observed between Activated mast cells and Monocytes (R = 0.75, Figure 9b). The correlation analysis between key genes and immune cells reveals a significant negative correlation between the expression of KLHDC3 and Follicular helper T cells, as well as Activated NK cells (*P* < 0.05, Figure 9c). The expression of NDRG1 is significantly positively correlated with Follicular helper T cells and Activated mast cells (*P* < 0.05), and significantly negatively correlated with Macrophages M0 and Resting mast cells (*P* < 0.05, Figure 9d).

### 3.9 Drug candidate prediction and molecular docking

**Figure 10:**
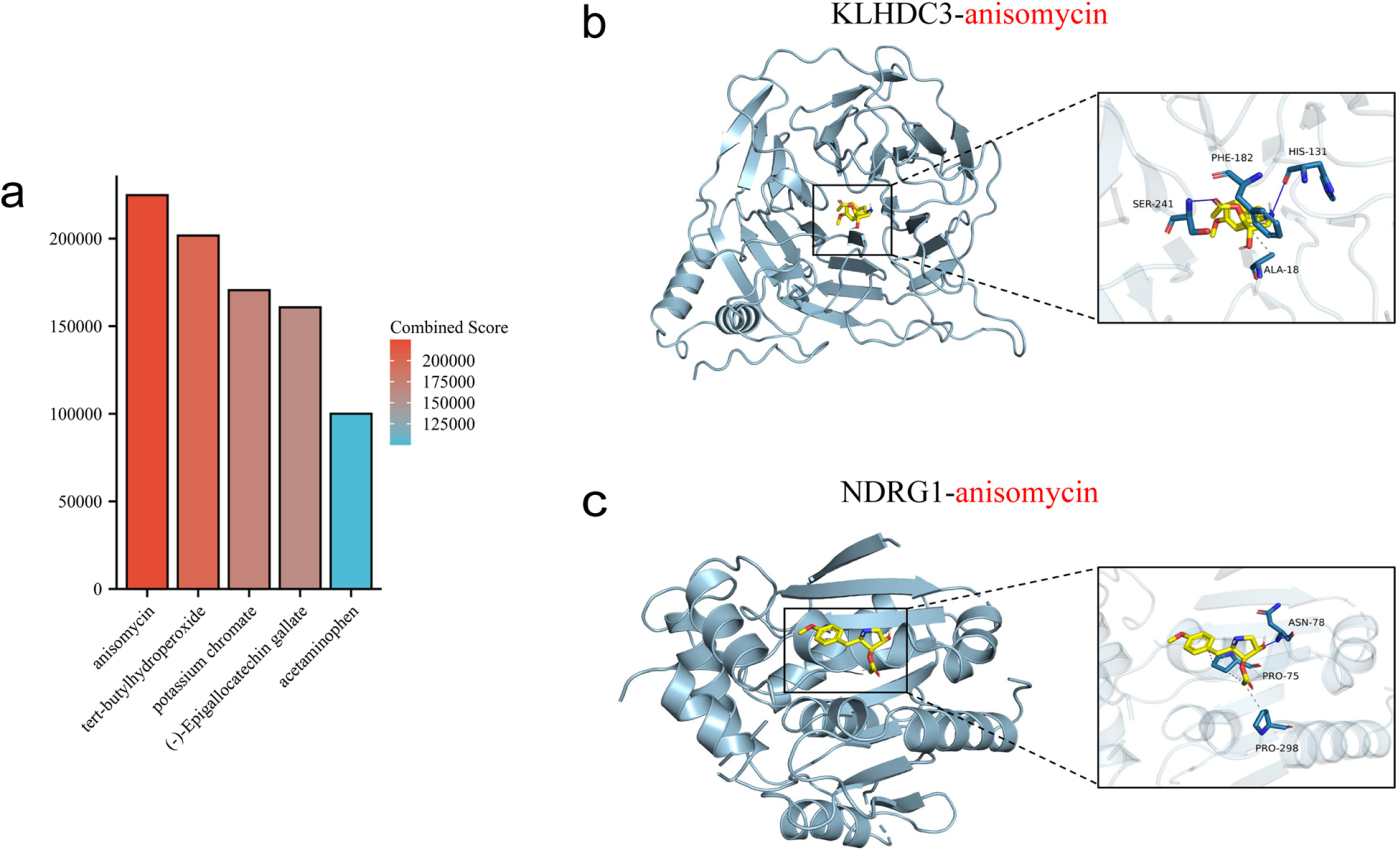
Prediction and molecular docking of candidate drugs. **(a)** Top five ranked small-molecule drugs. **(b,c)** Molecular docking of anisomycin with KLHDC3 and NDRG1.

The top 5 ranked compounds are anisomycin, tert-butylhydroperoxide, potassium chromate, (-)-Epigallocatechin gallate and acetaminophen (Figure 10a). The results indicate that anisomycin is the most promising small molecule drug for treating DIC. We further used molecular docking to validate the relative binding affinity of anisomycin with KLHDC3 (−6.8 kcal/mol) and NDRG1 (−4.8 kcal/mol) (Figures 10b,c).

## 4 Discussion

DOX significantly improves the survival and prognosis of cancer patients, but it also induces corresponding cardiotoxic side effects. Approximately 10% of patients undergoing DOX treatment may develop cardiovascular complications post-drug discontinuation, with the occurrence probability closely tied to dosage (7). Clinically, DIC typically presents as reduced ventricular ejection fraction and symptoms like pericarditis and heart failure, exacerbating the patient's condition and potentially leading to treatment interruption or death in severe cases (13, 14). DOX induces left ventricular systolic dysfunction and heart failure in a dose-dependent manner, with echocardiography, electrocardiography, biomarker detection and cardiac imaging effectively reflecting the severity of myocardial damage. For patients with DIC, cardiovascular toxicity risk assessment should be conducted prior to tumor diagnosis and the initiation of treatment, and individualized cardiovascular risk management strategies should be further adopted, so as to achieve the goal of improving the quality of life and survival rate of DIC patients (15). The baseline data for cardiovascular risk assessment primarily includes age, gender, race, history of cardiovascular disease and treatment plans, lifestyle. Primary or secondary prevention strategies should be considered for patients with a history of cardiovascular disease and cancer therapy-related cardiovascular toxicity. Currently, the ESC (European Society of Cardiology) guidelines recommend that Minimize the use of cardiotoxic drugs, ACE-I/ARB and beta-blockers, Dexrazoxane/liposomal anthracyclines and Statins as general preventive strategies for high-risk patients to mitigate cardiovascular toxicity (16). A double-blind randomized trial by Tashakori et al. found that daily administration of carvedilol at 6.25 mg during chemotherapy in breast cancer patients could prevent the occurrence of DIC (17). Meanwhile, research by Lee et al. indicated that concomitant administration of low-dose candesartan with DOX-containing chemotherapy might be effective in preventing an early decrease in left ventricular ejection fraction among breast cancer patients without cardiovascular risk (18).

The mechanism of cardiotoxicity induced by various anti-tumor drugs is closely related to ferroptosis (19). Chen et al. discovered that DOX-induced cardiac ferroptosis in mice is associated with the upregulation of Hmox1, and targeted delivery of siHmox1 into cardiomyocytes could block DOX-induced ferroptosis and subsequent cardiotoxicity, presenting a potential therapeutic strategy for DIC (20). The cellular repressor of E1A-stimulated genes 1 (CREG1) is a cardioprotective factor. Liu et al. demonstrated through *in vivo* and *in vitro* experiments that overexpression of CREG1 could inhibit ferroptosis in cardiomyocytes, thereby alleviating DIC (21). Zhu et al. established models of DIC in cardiomyocytes and mice, verifying that the disruption of histamine/H_1_R-STAT3-SLC7A11 axis could trigger ferroptosis and exacerbate DIC (22). Additionally, etoposide activated the p53-mediated ferroptosis pathway, which may be a key mechanism underlying its cardiotoxic effects (23). Research on DIC mechanisms has intensified (7, 24), with ferroptosis emerging as a novel cell death mode (25, 26). Further investigation into FRGs in DIC can illuminate its pathogenesis and underpin preventive and therapeutic strategies.

In this study, we initially retrieved the gene chip dataset of DOX and CON from the GEO database, resulting in 1,380 differential genes identified through merging analysis. Subsequent gene enrichment analysis revealed notable upregulation in biological processes, including Natural Killer cell-mediated cytotoxicity, Diseases of Metabolism, Lysosome function, Glycosaminoglycan Metabolism, and Extracellular Matrix Degradation. Conversely, pathways including Signaling by RHO GTPases, Regulation of Lipid Metabolism by PPARalpha, RNA Metabolism, Cell Cycle, and Cell Cycle Checkpoints were notably downregulated.

We acquired FRGs from Genecards, FerrDb V2, Geneontology and GSEA databases, resulting in 119 genes after intersection with DEGs. Enrichment analysis revealed that these intersecting genes are primarily associated with oxidative stress response and cellular response to chemical stress in terms of BP. Regarding CC, they are linked with the endoplasmic reticulum, collagen-containing extracellular matrix, and methyltransferase. Concerning MF, they exhibit associations with ubiquitin-protein ligase, DNA-binding transcription factors, and transcription coactivator activity. Regarding KEGG pathways, these genes are primarily enriched in mitosis, ferroptosis, cell apoptosis, NOD-like receptor pathway, Apelin signaling pathway, and TGF-β signaling pathway. Therefore, we hypothesize that ferroptosis may contribute to the onset and progression of DIC via oxidative stress, inflammatory response and apoptosis.

We identified the key genes of DE-FRGs as KLHDC3, NDRG1 and SPHK1 by using LASSO regression, SVM-RFE algorithm and RF algorithm. Meanwhile, external datasets were used for validation, and the results showed statistically significant differences in the expression of KLHDC3 and NDRG1 between the DOX and CON groups, while SPHK1 expression exhibited no statistical significance between the two groups. Therefore, in subsequent experiments, we only validated the expression levels of KLHDC3 and NDRG1. Additionally, ROC curves were plotted to validate the high diagnostic value of KLHDC3 and NDRG1. By establishing a H9c2 cell damage model, we utilized qRT-PCR to validate the expression levels of key genes. The results indicated significant differences in the expression levels of KLHDC3 and NDRG1 between the DOX and CON groups. Overexpression of ferroptosis markers glutathione peroxidase 4 (GPX4) and solute carrier family 7 member 11 (SLC7A11) has been shown to significantly ameliorate DOX-induced myocardial injury (27). Han et al. found that calycosin can alleviate the condition by upregulating the expression levels of GPX4 and SLC7A11 in patients with DIC (28). Wang et al. demonstrated that DOX promotes ferroptosis and myocardial lipid peroxidation by downregulating GPX4 and upregulating acyl-CoA synthetase long-chain family member 4, thereby exacerbating cardiac injury (29). Our study explores novel biomarkers KLHDC3 and NDRG1 for DIC, providing additional strategies for the treatment of DIC patients.

Target miRNAs of KLHDC3 included hsa-miR-24-3p, hsa-miR-486-3p and hsa-miR-214-3p, while those of NDRG1 comprised hsa-miR-4510, hsa-miR-182-5p and hsa-miR-96-5p. The study found that hsa-miR-24-3p plays an important role in the occurrence and development of cancer (30). Following DOX treatment of mouse cardiomyocytes, miR-24-3p expression significantly decreased, thereby mitigating DOX-induced myocardial injury primarily through oxidative stress reduction (31). Research has demonstrated that hsa-miR-486-3p regulates EGFR expression by inducing apoptosis, impacting cancer cell drug resistance. Moreover, it serves as a stable biomarker in acute coronary syndromes (32, 33). Another study reported upregulation of hsa-miR-486-3p expression in DOX-treated cardiomyocytes, exacerbating cardiac dysfunction and ultimately leading to cardiomyopathy (34). Research on ovarian cancer revealed that hsa-miR-214-3p downregulation inhibited its progression and could contribute to triple-negative breast cancer development through regulation of cell invasion, metastasis and proliferation (35, 36). Research indicated a significant role for miR-4510 in gastric and liver cancer occurrence and development (37, 38). Recent studies have foud that hsa-miR-182-5p and hsa-miR-96-5p are both up-regulated in the expression level of DOX-induced cardiomyocytes, which leads to cardiac damage, and both of them can be used as potential markers of drug-induced cardiotoxicity (33, 39). lncRNAs competitively bind to the 3’-UTR of target gene mRNA with miRNAs, exerting a negative regulatory effect by inhibiting miRNA action on the target gene. Subsequently, we identified target lncRNAs for the mentioned miRNAs and confirmed the relationship by selecting lncRNAs with CLIP-Data >5 and employing Sankey diagrams. Recent studies have drawn attention to the significant role of lncRNAs in drug-associated cardiotoxicity, with NEAT1 exhibiting cardioprotective effects via the miR-142-3p/FOXO1 signaling pathway. Moreover, it inhibits miR-221-3p expression to mitigate DOX-induced cellular senescence (40, 41). XIST promotes cardiovascular disease progression by regulating endothelial dysfunction, inflammation and oxidative stress. Dysregulation of XIST is closely associated with atherosclerosis, hypertrophic cardiomyopathy and myocardial fibrosis (42). A study revealed that lncRNA-MALAT1/miR-92a-3p/ATG4a-mediated exosomal hypoxia conferred protection against DOX-induced cardiac injury, suggesting exosomal hypoxia as a potential therapeutic target for DIC (43). SNHG1 mitigates myocardial ischemia-reperfusion injury by modulating the miR-137-3p/KLF4/TRPV1 axis and attenuates hypoxia-reoxygenation-induced vascular endothelial cell injury via the HIF-1*α*/VEGF signaling pathway (44, 45). SNHG16 accelerates atherosclerosis progression through the miRNA-22-3p/HMGB2 axis, and it also promotes abdominal aortic aneurysm progression through miR-106b-5p/STAT3 (46, 47). In this study, we identified many relevant targets by constructing the KLHDC3/NDRG1-miRNA-lncRNA network, and we expect that these targets can be validated in the future, which will provide some insights for preventing and ameliorating DIC progression.

Analysis of immune cell infiltration reveals that Follicular helper T cells, Macrophages M0 and Resting mast cells are implicated in DIC pathogenesis. The correlation analysis establishing the relationship between key genes and immune cells confirms the association of KLHDC3 and NDRG1 with immune cells. The immune response significantly influences the onset and progression of cardiovascular diseases (48). Studies show an increase in M1 macrophage expression and significant suppression of M2 macrophages in the DIC mouse model (49, 50). Further investigation is necessary to elucidate the specific mechanisms underlying the involvement of immune cells in DIC.

DSigDB is a database that links gene expression profiles with drugs and molecular compounds for drug repurposing and research. This study predicted drug candidates based on KLHDC3 and NDRG1, revealing that the top 5 compounds, ranked by composite score and adjusted *P*-value, included anisomycin, tert-butylhydroperoxide, potassium chromate, (-)-Epigallocatechin gallate and acetaminophen. The predictions indicate anisomycin as the most promising drug for improving DIC, supported by molecular docking results showing its strong binding ability. Anisomycin, isolated by Pfizer in 1954 from Streptomyces roseochromogenes and Streptomyces griseolus fermentation broth, selectively binds to the 60S ribosomal subunit, inhibiting peptide bond formation. It exhibits various excellent biological and pharmacological activities, including anticancer and immunosuppressive effects. During myocardial ischemia-reperfusion, anisomycin activates c-Jun N-terminal kinase (JNK), contributing to myocardial cell apoptosis and inflammatory response (51, 52). Research has demonstrated that anisomycin inhibits ovarian cancer cell activity by reducing ATP and glutathione levels, while increasing lipid oxidation, malondialdehyde and Fe^2+^ levels. It also modulates the p38 MAPK pathway and H3S10 phosphorylation to promote ferroptosis (53, 54). A study on liver cancer found that anisomycin not only exerted a direct cytotoxic effect on hepatocellular carcinoma but also enhanced natural killer cell-mediated immunotherapy by increasing the expression of immunoregulatory genes (55). KLHDC3, a member of the kelch family, demonstrates heightened expression in non-small cell lung cancer, suggesting unfavorable patient outcomes. Moreover, *in vitro* and *in vivo* experiments confirm its role in regulating tumor growth and ferroptosis through p14ARF-NRF2-SLC7A11 (56–58). NDRG1, a member of the NDRG family, shows increased expression following treatment with iron chelating agents and in cancer cells (59). DIC was found to disrupt iron metabolism, with DOX-treated cells exhibiting increased NDRG1 mRNA expression (60). Hence, anisomycin is speculated to be a promising candidate for future DIC treatment.

However, our study relies solely on H9c2 and hiPSC-CMs, which do not fully replicate primary human cardiomyocytes. For instance, H9c2 cells lack T-tubules, which play a critical role in human cardiac cells, and hiPSC-CMs differ greatly in the maturation state of their electrophysiological properties. Therefore, future studies could incorporate primary cardiomyocytes or further *in vivo* experimental validation. Additionally, we look forward to validating the expression levels of KLHDC3 and NDRG1 in broader datasets. Furthermore, we predict that anisomycin may be a potential therapeutic agent for DIC, and its therapeutic potential should be further explored through experimental studies in the future.

This study draws conclusions based on bioinformatics analysis and experiment validation with some limitations: gene-expression datasets which are publicly available, may have inherent biases (sample size, population specificity, data quality); lack of molecular dynamics simulation and experimental validation for predicted drug and key genes. In the future, we will continue to expand the sample size and conduct experimental validation to enhance the reliability of the study.

## 5 Conclusion

Ferroptosis is involved in the pathogenesis of DIC. We screened and validated the biomarkers of ferroptosis associated with DIC as KLHDC3 and NDRG1 based on bioinformatics, further analyzed their diagnostic value, constructed the miRNA-lncRNA network and evaluated the correlation with immune cells. In addition, drug prediction suggested that anisomycin may be a potential therapeutic agent for DIC. These findings provide new theoretical support for the diagnosis and treatment of DIC.

## Data Availability

The original contributions presented in the study are included in the article/Supplementary Material, further inquiries can be directed to the corresponding author.
