# Supplementary material for: Bioinformatics analysis of ferroptosis-related biomarkers and potential drug predictions in doxorubicin-induced cardiotoxicity
Source: Front Cardiovasc Med. 2025 Apr 24;12:1566782. doi: 10.3389/fcvm.2025.1566782 (PMC12058674; doi:10.3389/fcvm.2025.1566782)
Supplement: Supplementary file 1 [file Datasheet1.zip › R code.docx]

#Data processing

library(tidyr)

library(sva)

library(limma)

library(dplyr)

library(impute)

library(tibble)

library(factoextra)

com = function(dir,pa1,m,n,pa2) {

gse = data.frame()

for (f in list.files(dir)) {

if (length(grep(pa1,f)) == 1) {

print(f)

d = read.delim(paste0(dir,f))

d = d[,c(m,n)]

colnames(d) = c('ENSG','count')

d$count = as.numeric(d$count)

d = aggregate(count~ENSG,d,median)

colnames(d)[2] = strsplit(f,'_')[[1]][1]

if (ncol(gse) == 0) {gse = d} else {

gse = cbind(gse,d)

}

}

}

rownames(gse) = gse$ENSG

gse = gse[,grep(pa2,colnames(gse))]

return(gse)

}

gene = read.delim('../gene_exons_len.txt',header=F)

gene = separate(gene,1,c('ensg',NA),'\\.')

bed = read.delim('../gencode.v42lift37.sort.bed',header=F)

bed = separate(bed,4,c('ensg','gene','entrez','c'),'\\|')

cf = function(exp,i,first,GSE) {

counts2fpkm = function(counts, effLen){

N = sum(counts)

fpkm = exp( log(counts) + log(1e9) - log(effLen) - log(N) )

return(fpkm)

}

id3 = match(rownames(exp),bed[,i])

exp = exp[-which((is.na(id3))),]

id4 = match(bed[na.omit(id3),4],gene[,1])

genel = gene[id4,]

exp = apply(exp, 2, counts2fpkm,effLen=genel[,2])

exp = log2(exp+1)

if (!(is.na(as.numeric(rownames(exp)[1])))) {

rownames(exp) = bed[na.omit(id3),5]

} else if (length(grep('ENSG',rownames(exp)[1])) == 1) {

rownames(exp) = bed[na.omit(id3),5]

}

exp = as.data.frame(exp)

exp$gene = rownames(exp)

exp = exp %>% filter(gene %in% rownames(first))

write.table(exp[,-ncol(exp)],paste0(GSE,'_logFPKM.txt'),row.names = T,col.names = T,sep = '\t',quote = F)

return(exp)

}

first = read.delim('../combined_disease.txt')

g861 = com('GSE193861/','txt$',1,2,'GSM')

g861 = g861[-(1:5),]

g861 = cf(g861,4,first,'GSE193861')

g803 = read.delim('GSE206803人来源的心肌细胞及鼠/GSE206803_iPSC-CM_raw_counts.txt')

rownames(g803) = g803[,1]

col = colnames(g803)[1:22]

g803 = g803[,-1]

colnames(g803) = col

g803 = round(g803)

g803 = cf(g803,4,first,'GSE206803')

g674 = read.table('GSE243674人来源的心肌细胞/GSE243674_Counts_RNA_ERMatthews.txt',sep=' ',row.names = 1,header = T)

g674 = cf(g674,6,first,'GSE243674')

g470 = read.delim('GSE235470人来源心肌细胞/GSE235470_rawcounts.counts.txt')

g470 = g470[!duplicated(g470[,10]),]

rownames(g470) = g470[,10]

g470 = g470[,2:9]

g470 = cf(g470,5,first,'GSE235470')

inter = g803 %>% inner_join(g470,by='gene') %>%

inner_join(g674,by='gene')

inter = tibble::column_to_rownames(inter,var = 'gene')

batch = c(rep('batchA',ncol(g803)-1),rep('batchB',ncol(g470)-1),rep('batchC',ncol(g674)-1))

combat_data = ComBat(dat=as.matrix(inter), batch=batch)#change min value less than 0

combat_norm = normalizeQuantiles(combat_data)

write.table(combat_norm,'803_470_674_batch_norm.txt',row.names = T,col.names = T,sep='\t',quote = F)

probe2gene = function(gse_series_mtrx, skip_num, platform_anno, dir) {

anno = read.table(platform_anno,header=T,sep="\t",stringsAsFactors=F)

gse = read.table(paste(dir,gse_series_mtrx,sep='/'),sep="\t",skip=skip_num,header=T,row.names=1,stringsAsFactors=F,fill=T)

gse = gse[-nrow(gse),]

order = match(rownames(gse),anno$ID)

use_probe = which(is.na(order) == F)

gse = as.matrix(gse[use_probe,])

order = order[use_probe]

rownames(gse) = anno[order,2]

print(dim(gse))

return (gse)

}

process = function(gse) {

d1 = is.na(gse)

d2 = rep(0,dim(gse)[1])

for (i in 1:dim(d1)[1]) {

dd = length(which(d1[i,] == T))

d2[i] = dd

}

delete = which(d2>dim(gse)[2]/2)

if (length(delete)>0) gse = gse[-delete,]

Med = median(gse,na.rm = T)

if(Med > 16) {

gse = log2(gse) } else {print ("expression data has been logged")}

na.length = length(which(is.na(gse)==T))

if(na.length > 0) gse = impute.knn(gse)$data

print(dim(gse))

return (gse)

}

normalize = function(gse,merge,norm,dir,me='median') {

tmp = aggregate(gse,list(rownames(gse)),median)

#code = paste0('tmp=aggregate(',gse,',list(rownames(',gse,')),',me,')')

#eval(parse(text = code))

gse = as.matrix(tmp[,-1])

rownames(gse) = tmp[,1]

data = normalizeQuantiles(gse)

write.table(gse,paste(dir,merge,sep='/'),sep="\t",quote=F,row.names=T,col.names=T)

write.table(data,paste(dir,norm,sep='/'),sep="\t",quote=F,row.names=T,col.names=T)

print(dim(data))

}

indir = '.'

GSE40447_gene = probe2gene('GSE40447人全血/GSE40447_series_matrix.txt',67,'GSE40447人全血/GPL16006-7713.anno.txt',indir)

GSE40447_gene = process(GSE40447_gene)

normalize(GSE40447_gene,'GSE40447_gene.txt','GSE40447.txt','.')

g297d = read.delim('../GSE106297_disease.txt')

g297c = read.delim('../GSE106297_control.txt')

g297 = g297d %>% bind_cols(g297c) %>%

rownames_to_column(var='gene')

g282d = read.delim('../GSE157282_disease.txt')

g282c = read.delim('../GSE157282_control.txt')

cols = c('gene',"GSM4760744","GSM4760745","GSM4760746","GSM4760753","GSM4760754","GSM4760755")

g282 = g282c %>% bind_cols(g282d) %>%

rownames_to_column(var='gene')

colnames(g282) = cols

g803_bk = g803

colnames(g803) = c("GSM6263857","GSM6263858","GSM6263859","GSM6263860","GSM6263868","GSM6263869","GSM6263870","GSM6263871","GSM6263861","GSM6263862","GSM6263863","GSM6263872","GSM6263873","GSM6263874",

"GSM6263864","GSM6263865","GSM6263866","GSM6263867","GSM6263875","GSM6263876","GSM6263877","GSM6263878",'gene')

inter1 = g803 %>% inner_join(g297,by='gene') %>%

inner_join(g282,by='gene')

inter1 = tibble::column_to_rownames(inter1,var = 'gene')

batch = c(rep('batchA',ncol(g803)-1),rep('batchB',ncol(g297)-1),rep('batchC',ncol(g282)-1))

combat_data1 = ComBat(dat=as.matrix(inter1), batch=batch)#change min value less than 0

combat_norm1 = normalizeQuantiles(combat_data1)

write.table(combat_norm1,'803_297_282_batch_norm.txt',row.names = T,col.names = T,sep='\t',quote = F)

data_box = combat_norm1[,c(15:22,31:34,23:26,35:40)]

pdf('boxplot.pdf',width=14,height = 6)

par(oma=c(2,1,1,1))

boxplot(data_box,axes = F,outline=F,xlim=c(1,25),ylim=c(-0.5,8),ylab='Expression',col=c(rep('skyblue',8),rep('tomato',8),rep('yellowgreen',6)))

axis(2)

axis(1, at = 1:22,labels=colnames(data_box),las=2,pos=-0.8)

legend(22.5,8,col=c('skyblue','tomato','yellowgreen'),legend=c('GSE206803','GSE106297','GSE157282'),pch=c(15,15),box.lwd=0,ncol=1)

dev.off()

before = t(inter1[,c(15:26,31:40)])

after = t(combat_data1[,c(15:26,31:40)])

plot_pca = function(figname,data) {

pca = prcomp(data)

batchs = c(rep('GSE206803',8),rep('GSE106297',8),rep('GSE157282',6))

pdf(figname)

p = fviz_pca_ind(pca, label="none", habillage=batchs,

addEllipses=TRUE, ellipse.level=0.95,

palette = c("#E69F00", "#56B4E9",'#9ACD32'))

print(p)

dev.off()

}

plot_pca('before.pdf',before)

plot_pca('after.pdf',after)

#limma

library(limma)

library(dplyr)

a<-read.csv("datazui.csv",row.names = 1)

list <- c(rep("BA", 7), rep("DC",7)) %>% factor(., levels = c("BA", "DC"), ordered = F)

list <- model.matrix(~factor(list)+0) #把group设置成一个model matrix

colnames(list) <- c("BA", "DC")

df.fit <- lmFit(a, list) ## 数据与list进行匹配

df.matrix <- makeContrasts(BA - DC, levels = list)

fit <- contrasts.fit(df.fit, df.matrix)

fit <- eBayes(fit)

tempOutput <- topTable(fit,coef=1,n = Inf, adjust = "BH")

nrDEG = na.omit(tempOutput) ## 去掉数据中有NA的行或列

diffsig <- nrDEG

write.csv(diffsig, "all.limmaOut.csv")

## 我们使用|logFC| > 1，padj < 0.05（矫正后P值）

foldChange = 1

padj = 0.05

## 筛选出所有差异基因的结果

All_diffSig <- diffsig[(diffsig$adj.P.Val < padj & (diffsig$logFC>foldChange | diffsig$logFC < (-foldChange))),]

write.csv(All_diffSig, "all.diffsig.csv") ##输出差异基因数据集

diffup <- All_diffSig[(All_diffSig$adj.P.Val < padj & (All_diffSig$logFC > foldChange)),]

write.csv(diffup, "diffup.csv")

#

diffdown <- All_diffSig[(All_diffSig$adj.P.Val < padj & (All_diffSig$logFC < -foldChange)),]

write.csv(diffdown, "diffdown.csv")

#GSEA

library(tidyverse)

library(cowplot)

library(clusterProfiler)

library(enrichplot)

library(plyr)

library(ggrepel)

library(ggplot2)

library(RColorBrewer)

library(gridExtra)

library(limma)

library(dplyr)

a<-read.csv("datazui.csv",row.names = 1)

a<-as.data.frame(t(a))

a<-a[order(a$PDCD1),]

a<-as.data.frame(t(a))

list <- c(rep("high", 39), rep("low",39)) %>% factor(., levels = c("high", "low"), ordered = F)

list <- model.matrix(~factor(list)+0) #把group设置成一个model matrix

colnames(list) <- c("high", "low")

df.fit <- lmFit(a, list) ## 数据与list进行匹配

df.matrix <- makeContrasts(low - high, levels = list)

fit <- contrasts.fit(df.fit, df.matrix)

fit <- eBayes(fit)

tempOutput <- topTable(fit,coef=1,n = Inf, adjust = "BH")

nrDEG = na.omit(tempOutput) ## 去掉数据中有NA的行或列

diffsig <- nrDEG

write.csv(diffsig, "all.limmaOut.csv")

## 我们使用|logFC| > 0.5，padj < 0.05（矫正后P值）

foldChange = 1

padj = 0.05

## 筛选出所有差异基因的结果

All_diffSig <- diffsig[(diffsig$adj.P.Val < padj & (diffsig$logFC>foldChange | diffsig$logFC < (-foldChange))),]

write.csv(All_diffSig, "all.diffsig.csv") ##输出差异基因数据集

diffup <- All_diffSig[(All_diffSig$adj.P.Val < padj & (All_diffSig$logFC > foldChange)),]

write.csv(diffup, "diffup.csv")

#

diffdown <- All_diffSig[(All_diffSig$adj.P.Val < padj & (All_diffSig$logFC < -foldChange)),]

write.csv(diffdown, "diffdown.csv")

gsym.fc<-diffsig

gsym.fc$SYMBOL<-rownames(gsym.fc)

gsym.id <- bitr(rownames(diffsig), fromType = "SYMBOL", toType = "ENTREZID", OrgDb = "org.Hs.eg.db")

gsym.fc.id <- merge(gsym.fc, gsym.id, by="SYMBOL", all=F)

gsym.fc.id.sorted <- gsym.fc.id[order(gsym.fc.id$logFC, decreasing = T),]

id.fc <- gsym.fc.id.sorted$logFC

names(id.fc) <- gsym.fc.id.sorted$ENTREZID

kk <- gseKEGG(id.fc, organism = "hsa")

dim(kk)

#head(kk)

kk.gsym <- setReadable(kk, 'org.Hs.eg.db',

'ENTREZID')

sortkk <- kk.gsym[order(kk.gsym$enrichmentScore, decreasing = T),]

write.csv(sortkk,"gsea_output.csv", quote = F, row.names = F)

# 要画的通路

geneSetID <- c("hsa04742","hsa04740")

# 突出显示感兴趣的基因

selectedGeneID <- c("OR2A7")

# 自定义足够多的颜色

mycol <- c("darkgreen","chocolate4","blueviolet","#223D6C","#D20A13","#088247","#58CDD9","#7A142C","#5D90BA","#431A3D","#91612D","#6E568C","#E0367A","#D8D155","#64495D","#7CC767")

for (i in geneSetID) {

gseaplot(kk, i)

myGeneList <- enrichplot:::gsInfo(kk, i)

row.names(myGeneList) <- gsym.fc$gsym

myGeneList$id <- gsym.fc$ENTREZID

write.csv(myGeneList, paste0("gsea_genelist_", i, "_group1.csv"))

}

x <- kk

geneList <- position <- NULL ## to satisfy codetool

#合并多条通路的数据

gsdata <- do.call(rbind, lapply(geneSetID, enrichplot:::gsInfo, object = x))

gsdata$gsym <- rep(gsym.fc.id.sorted$SYMBOL,2)

# 画running score

p.res <- ggplot(gsdata, aes_(x = ~x)) + xlab(NULL) +

geom_line(aes_(y = ~runningScore, color= ~Description), size=1) +

scale_color_manual(values = mycol) +

#scale_x_continuous(expand=c(0,0)) + #两侧不留空

geom_hline(yintercept = 0, lty = "longdash", lwd = 0.2) + #在0的位置画虚线

ylab("Enrichment\n Score") +

theme_bw() +

theme(panel.grid = element_blank()) + #不画网格

theme(legend.position = "top", legend.title = element_blank(),

legend.background = element_rect(fill = "transparent")) +

theme(axis.text.y=element_text(size = 12, face = "bold"),

axis.text.x=element_blank(),

axis.ticks.x=element_blank(),

axis.line.x=element_blank(),

plot.margin=margin(t=.2, r = .2, b=0, l=.2, unit="cm"))

#p.res

# 画rank

rel_heights <- c(1.5, .5, 1.5) # 上中下三个部分的比例

i <- 0

for (term in unique(gsdata$Description)) {

idx <- which(gsdata$ymin != 0 & gsdata$Description == term)

gsdata[idx, "ymin"] <- i

gsdata[idx, "ymax"] <- i + 1

i <- i + 1

}

#head(gsdata)

p2 <- ggplot(gsdata, aes_(x = ~x)) +

geom_linerange(aes_(ymin=~ymin, ymax=~ymax, color=~Description)) +

xlab(NULL) + ylab(NULL) +

scale_color_manual(values = mycol) + #用自定义的颜色

theme_bw() +

theme(panel.grid = element_blank()) + #不画网格

theme(legend.position = "none",

plot.margin = margin(t=-.1, b=0,unit="cm"),

axis.ticks = element_blank(),

axis.text = element_blank(),

axis.line.x = element_blank()) +

#scale_x_continuous(expand=c(0,0)) +

scale_y_continuous(expand=c(0,0))

#p2

# 画变化倍数

df2 <- p.res$data

df2$y <- p.res$data$geneList[df2$x]

df2$gsym <- p.res$data$gsym[df2$x]

#head(df2)

# 提取感兴趣的基因的变化倍数

selectgenes <- data.frame(gsym = selectedGeneID)

selectgenes <- merge(selectgenes, df2, by = "gsym")

selectgenes <- selectgenes[selectgenes$position == 1,]

head(selectgenes)

p.pos <- ggplot(selectgenes, aes(x, y, fill = Description, color = Description, label = gsym)) +

geom_segment(data=df2, aes_(x=~x, xend=~x, y=~y, yend=0),

color = "grey") +

geom_bar(position = "dodge", stat = "identity") +

scale_fill_manual(values = mycol, guide=FALSE) + #用自定义的颜色

scale_color_manual(values = mycol, guide=FALSE) + #用自定义的颜色

#scale_x_continuous(expand=c(0,0)) +

geom_hline(yintercept = 0, lty = 2, lwd = 0.2) + #在0的位置画虚线

ylab("Ranked list\n metric") +

xlab("Rank in ordered dataset") +

theme_bw() +

theme(axis.text.y=element_text(size = 12, face = "bold"),

panel.grid = element_blank()) +

# 显示感兴趣的基因的基因名

geom_text_repel(data = selectgenes,

show.legend = FALSE, #不显示图例

direction = "x", #基因名横向排列在x轴方向

ylim = c(2, NA), #基因名画在-2下方

angle = 90, #基因名竖着写

size = 2.5, box.padding = unit(0.35, "lines"),

point.padding = unit(0.3, "lines")) +

theme(plot.margin=margin(t = -.1, r = .2, b=.2, l=.2, unit="cm"))

#p.pos

# 组图

plotlist <- list(p.res, p2, p.pos)

n <- length(plotlist)

plotlist[[n]] <- plotlist[[n]] +

theme(axis.line.x = element_line(),

axis.ticks.x = element_line(),

axis.text.x = element_text(size = 12, face = "bold"))

plot_grid(plotlist = plotlist, ncol = 1, align="v", rel_heights = rel_heights)

ggsave("GSEA_multi_pathways.pdf", width=6, height=5)

# LASSO

library(tibble)

library(tidyverse)

library(broom)

library(glmnet)

df<-read.csv("expzui.csv",row.names = 1)

k_transposed <- as.data.frame(t(k))#行列转置

k<-read.csv("expzui.csv",row.names = 1)

k_transposed <- as.data.frame(t(k))#行列转置

a<-k_transposed

gene<-read.csv("31gene.csv")

k<-a[rownames(a)%in%gene$gene,]

k<-as.data.frame(t(k))

fen<-read.csv("fen.csv",row.names = 1)

k<-k[rownames(fen),]

k<-cbind(fen$lasso,k)

states<-as.matrix(k)

x<-states[,-1]

y<-states[,1]

cvfit=cv.glmnet(x,y,type.measure = "mse",nfolds = 5,alpha=1)

plot(cvfit)

cvfit$lambda.min

c(cvfit$lambda.min, cvfit$lambda.1se)

lasso<-glmnet(x,y,family="binomial",alpha=1,nlambda = 100)

coef(lasso, s=c(0.00352157,0.01879356))#根据lambda.min,lambda.1se数值进行更改。

print(lasso) #结果中依次特征数，偏差解释比，参数λ，通常看最后一

plot(lasso,label=T) #L1范数

plot(lasso,xvar="lambda",label=T) #lambda参数

lasso.coef<-predict(lasso,s=0.4,type="coefficients") #回归系数

plot(lasso,xvar="dev",label=T) #解释偏差和回归系数的关系

lasso.y<-predict(lasso,newx=x,type="response",s=0.4) #拟合

plot(lasso.y,y,xlab="Predicted",ylab="Actual",main="Lasso Regression")

#SVM-RFE

a<-read.csv("k.csv",row.names = 1)

library(tidyverse)

library(glmnet)

source('msvmRFE.R') #文件夹内自带

library(VennDiagram)

library(sigFeature)

library(e1071)

library(caret)

library(randomForest)

#library(e1071)

#source(msvmRFE.R)

train<-a

input <- train

#采用五折交叉验证 (k-fold crossValidation）

svmRFE(input, k = 5, halve.above = 100) #分割数据，分配随机数

nfold = 5

nrows = nrow(input)

folds = rep(1:nfold, len=nrows)[sample(nrows)]

folds = lapply(1:nfold, function(x) which(folds == x))

results = lapply(folds, svmRFE.wrap, input, k=5, halve.above=100) #特征选择

top.features = WriteFeatures(results, input, save=F) #查看主要变量

head(top.features)

#把SVM-REF找到的特征保存到文件

write.csv(top.features,"feature_svm.csv")

# 运行时间主要取决于选择变量的个数，一般的电脑还是不要选择太多变量

# 选前48个变量进行SVM模型构建，体验一下

featsweep = lapply(1:48, FeatSweep.wrap, results, input) #48个变量

featsweep

#load("featsweep.RData")

# 选前300个变量进行SVM模型构建，然后导入已经运行好的结果

#featsweep = lapply(1:300, FeatSweep.wrap, results, input) #300个变量

save(featsweep,file = "featsweep.RData")

#画图

no.info = min(prop.table(table(input[,1])))

errors = sapply(featsweep, function(x) ifelse(is.null(x), NA, x$error))

#dev.new(width=4, height=4, bg='white')

#pdf("B_svm-error.pdf",width = 5,height = 5)

PlotErrors(errors, no.info=no.info) #查看错误率

#dev.off()

#dev.new(width=4, height=4, bg='white')

#pdf("B_svm-accuracy.pdf",width = 5,height = 5)

Plotaccuracy(1-errors,no.info=no.info) #查看准确率

#dev.off()

# 图中红色圆圈所在的位置，即错误率最低点

which.min(errors)

top<-top.features[1:which.min(errors), "FeatureName"]

write.csv(top,"top.csv")

#RF

library(caret)

library(shape)

display.progress = function (index, totalN, breakN=20) {

if ( index %% ceiling(totalN/breakN) ==0 ) {

cat(paste(round(index*100/totalN), "% ", sep=""))

}

}

k<-read.csv("k.csv",row.names = 1)

set.seed(19991018) # 设置种子

control <- rfeControl(functions = rfFuncs, # 选择随机森林；详细可参考http://topepo.github.io/caret/recursive-feature-elimination.html#rfe

method = "LGOCV", # 选择交叉验证法

number = 10) # 10折交叉验证

tmp <- k

candidate.gene<-colnames(k)

results <- rfe(x = tmp[,-1],

y =as.factor(k$fen.lasso),

metric = "Accuracy",

sizes = 1:(length(candidate.gene)-2), # 步长为1，速度较慢请耐心（约2小时）

rfeControl = control)

final.gene <- predictors(results) # 取出最终基因

write.table(final.gene,"output_selected features.txt",sep = "\t",row.names = F,col.names = F,quote = F)

accres <- results$results # 取出迭代结果

write.table(accres, "output_accuracy result.txt", sep = "\t", row.names = F,col.names = T,quote = F)

# 设置颜色

jco <- c("#2874C5","#EABF00")

# 图1：随机森林准确性图

pdf(file = "accuracy.pdf", width = 6, height = 4.5)

par(bty="o", mgp = c(2,0.5,0), mar = c(3.1,4.1,2.1,2.1),tcl=-.25,las = 1)

index <- which.max(accres$Accuracy) # 取出准确率最大时的索引（基因个数）

## 画圈圈

plot(accres$Variables,

accres$Accuracy,

ylab = "",

xlab = "Number of genes",

col = "steelblue")

## 添加连线

lines(accres$Variables,accres$Accuracy,col = "steelblue")

## 定位最大值

points(index, accres[index,"Accuracy"],

col = "steelblue",

pch = 19,

cex = 1.2)

## 补Y轴坐标（在plot时候写会和axis文字重叠）

mtext("Accuracy (Repeated Cross-Validation)",side = 2,line = 2.5, las = 3)

## 添加好看滴箭头

Arrows(x0 = index-7, x1 = index-2,

y0 = accres[index,"Accuracy"], y1 = accres[index,"Accuracy"],

arr.length = 0.2,

lwd = 2,

col = "black",

arr.type = "triangle")

## 添加基因数目信息

text(x = index - 7,

y = accres[index,"Accuracy"],

labels = paste0("N=",index),

pos = 2)

invisible(dev.off())

# sanky

library(readxl)

library(ggalluvial)

data_sanky = read_excel('桑基图重新.xlsx')

data_sanky = as.data.frame(data_sanky)

pdf('sanky.pdf',height=10)

p = ggplot(data = data_sanky,aes(axis1 = `Chinese herbalcompounds`,axis2 = Ingredients))+

scale_x_discrete(limits = c("Chinese herbalcompounds","Ingredients"),expand = c(0.3, 0.8))+

geom_alluvium(aes(fill = Ingredients)) +

geom_stratum(color="skyblue",alpha=.7) +

geom_text(stat ="stratum",aes(label = after_stat(stratum)),size=2) + #check_overlap = TRUE

guides(fill =FALSE) +

theme_bw() +

theme(panel.grid =element_blank()) +

theme(panel.border = element_blank()) +

theme(axis.line = element_blank(),axis.ticks =element_blank(),axis.text.y =element_blank())

plot(p)

dev.off()

# Immune infiltration analysis

library(tidyr)

library(reshape2)

head(a)

a$X<-rownames(a)

fen<-read.csv("fen.csv")

a<-merge(fen,a,"X")

mydata1<-melt(

a,

id.vars=c("X","fen"),

variable.name="immunecell",

value.name="tpm"

)

library(ggpubr)

library(ggplot2)

ylabname <- paste("immunecell", "expression")

colnames(mydata1) <- c("Sample", "Groups", "immunecell","tpm")

# 计算p value

pvalues <- sapply(mydata1$immunecell, function(x) {

res <- wilcox.test(as.numeric(tpm) ~ Groups, data = subset(mydata1, immunecell == x)) #两组，wilcox.test或t.test；多组，kruskal.test或aov(one-way ANOVA test)

res$p.value

})

pv <- data.frame(gene = mydata1$immunecell, pvalue = pvalues)

pv$sigcode <- cut(pv$pvalue, c(0,0.0001, 0.001, 0.01, 0.05, 1),

labels=c('****','***', '**', '*', 'ns'))

mydata1<-mydata1[,-1]

# 画box plot

p.box <- ggplot(mydata1, aes(x=immunecell, y=tpm, color=Groups, fill=Groups)) +

geom_boxplot(alpha = .5) + #半透明

theme_classic() + #或theme_bw()

scale_fill_brewer(palette = "Set1") + #按类填充颜色

scale_color_brewer(palette = "Set1") + #按类给边框着色

theme(axis.text.x = element_text(colour="black", size = 11,

#名太挤，旋转45度

angle = 45, hjust = .5, vjust = .5)) +

geom_text(aes(x=gene, y=max(mydata1$tpm) * 1.1,

label = pv$sigcode),

data=pv,

inherit.aes=F) +

ylab(ylabname)

p.box

ggsave("immunecellbox.pdf", width = 14, height = 5)

# 画带散点的box plot

p.box.dot <- p.box + geom_point(shape = 21, size=.5, # 点的形状和大小

position = position_jitterdodge(), # 让点散开

alpha = .5) #半透明

p.box.dot

ggsave("immunecellsanbox.pdf", width = 14, height = 5)
